# A Significant Decrease in the Incidence of Shigellosis in Israel during COVID-19 Pandemic

**DOI:** 10.3390/ijerph18063070

**Published:** 2021-03-16

**Authors:** Ravit Bassal, Lital Keinan-Boker, Dani Cohen

**Affiliations:** 1 Israel Center for Disease Control, Ministry of Health, Sheba Medical Center, Ramat Gan 5262160, Israel; Lital.Keinan2@MOH.GOV.IL; 2 Department of Epidemiology and Preventive Medicine, Sackler Faculty of Medicine, School of Public Health, Tel Aviv University, Tel Aviv 6997801, Israel; dancohen@tauex.tau.ac.il; 3 School of Public Health, University of Haifa, Haifa 3498838, Israel

**Keywords:** COVID-19, outbreak, trend, enteric diseases, incidence

## Abstract

Severe Acute Respiratory Syndrome Coronavirus 2 (SARS-CoV-2) causes COVID-19 and is mostly person-to-person transmitted through respiratory droplets. The implications of the strategies implemented to prevent COVID-19 transmission on other infectious diseases are unclear. We aimed to appraise trends in the incidence of salmonellosis, shigellosis and campylobacteriosis in Israel during COVID-19 pandemic. Positive stool samples for *Salmonella*, *Shigella* and *Campylobacter* are reported on a monthly basis to the Israel Center for Disease Control from sentinel laboratories, within the framework of a surveillance network of bacterial culture-proven enteric diseases. Age-adjusted incidence rates per 100,000 of shigellosis, salmonellosis and campylobacteriosis were calculated. Mean rates before and after the local onset of COVID-19 pandemic in Israel were compared and Relative Risk Reduction (RRR) was calculated. Joinpoint was used to evaluate secular trends. The mean age-adjusted incidence rate of shigellosis in March–July 2020 was lower than the rate observed in March–July 2018–2019 (RRR = 86.6%), but also decreased for salmonellosis (RRR = 33.0%) and campylobacteriosis (RRR = 30.0%). Using Joinpoint we have shown that the decrease observed for shigellosis was significantly sharper (Annual Percent Change (APC) = −77.7) between February 2020 and May 2020 than for salmonellosis (APC = −14.0) between July 2019 and April 2020 and for campylobacteriosis (APC = −1.1) between January 2018 and July 2020. The preventive measures applied to reduce transmission of COVID-19, including social distancing and hand washing, were ecologically associated with a decreased risk of bacterial enteric diseases in Israel. The association was strongest for shigellosis, a disease that is mostly person-to-person transmitted, as compared to salmonellosis and campylobacteriosis which are mostly foodborne transmitted.

## 1. Introduction

Severe Acute Respiratory Syndrome Coronavirus 2 (SARS-CoV-2) causes COVID-19 and was first introduced in Wuhan, China, in December 2019. Since then, the virus spread rapidly in the world, and the first COVID-19 patient in Israel was diagnosed on 27 February 2020. Transmission of COVID-19 is person-to-person and can occur through respiratory droplets and direct contact with infected people [1,2,3].

Three elements were declared as the key safeguards in the prevention of COVID-19: social distancing, wearing masks and hand hygiene. The potential implications of these strategies implemented to prevent COVID-19 transmission on other infectious diseases are of interest.

The aim of the current study was to assess the temporal trends in the incidence of salmonellosis, shigellosis and campylobacteriosis in Israel during the COVID-19 outbreak. Our assumption was that there might be a general reduction in the culture-proven incidence of the three enteric diseases because of a decrease in visits to clinics and performance of stool cultures following episodes of diarrheal diseases in the community during the pandemic. We also hypothesized that the preventive measures adopted to reduce transmission of COVID-19, especially social distancing and hand washing, can also significantly contribute to the overall reduction in enteric disease incidence, albeit to a different extent corresponding to differences in their main modes of transmission.

## 2. Materials and Methods

The national sentinel enteric diseases laboratory-based network was established in 1997 by the Israel Center for Disease Control (ICDC), aiming to monitor the incidence of salmonellosis, shigellosis and campylobacteriosis in Israel. Positive stool samples for *Salmonella*, *Shigella* and *Campylobacter* of diarrhea patients are reported to the ICDC on a monthly basis from eight sentinel clinical microbiological laboratories as follows: Haemek Medical Center and Haifa Health Maintenance Organization (HMO) in the north; Sheba Medical Center, Maccabi Dan District HMO and Clalit Petah-Tikva HMO in central Israel; Hadassah Medical Centers and Meuhedet HMO in Jerusalem; and Soroka Medical Center in southern Israel. These laboratories are geographically distributed throughout the country and serve 50% of the Israeli population. The data collection methods were similar throughout the total collection period.

**Study design:** Temporal trends.

**Ethics**: The data collection by the national sentinel enteric diseases laboratory-based network was approved as part of the mandatory reporting of infectious diseases in Israel.

**Statistical analysis**: Monthly age-adjusted incidence rates were calculated per 100,000 from the data collected, using the 2008 Israeli population for adjustment. The 2008 Israeli Census population was used as a standard population for the comparison of secular trends in different time periods, since the Israeli population is young and has a non-negligible annual growth rate. The year 2008 was chosen since it represents nearly the mid-point of our follow-up. The mean age-adjusted incidence rate of salmonellosis, shigellosis and campylobacteriosis were calculated for the period March–July 2020 and were compared to March–July during 2018–2019, and the Relative Risk Reduction (RRR) was calculated (1-RR). We used SAS Enterprise Guide (version 7.12 (7.100.2.3350), SAS Institute Inc., Cary, NC, USA).

Secular trends in incidence were evaluated between January 2018 and July 2020 for salmonellosis, shigellosis and campylobacteriosis, using the Joinpoint software (Joinpoint Regression Program, Version 4.8.0.1, April 2020; Statistical Research and Applications Branch, National Cancer Institute). The slope between the best-fitting points (joinpoints) of the modeled age-adjusted rates were described by an Annual Percent Change (APC). Significance was declared at *p*-value < 0.05.

## 3. Results

**Figure 1:**
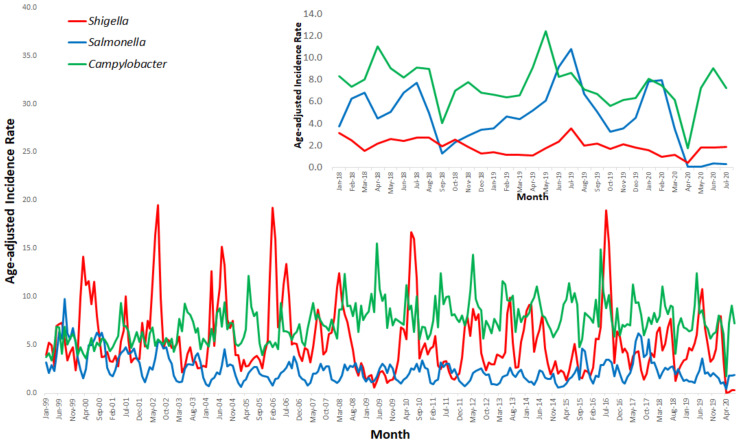
Salmonellosis, shigellosis and campylobacteriosis age-adjusted incidence rate per 100,000 in sentinel laboratories, Israel, January 1999–July 2020, and a focus on January 2018–July 2020.

The monthly age-adjusted incidence rates of salmonellosis, shigellosis and campylobacteriosis between January 1999 and July 2020 are presented in Figure 1. Figure 1 also presents a focused, monthly age-adjusted incidence rates between January 2018 and July 2020. 

The mean age-adjusted incidence rate of shigellosis was 0.9 (95% CI: 0.0–2.7) per 100,000 during March–July 2020, lower than the corresponding rate for March–July 2018–2019: 6.7 (95% CI: 5.2–8.2) per 100,000, and the RRR between the periods was 86.6% (Table 1). The mean age-adjusted incidence rate per 100,000 of salmonellosis and campylobacteriosis also decreased after the onset of the COVID-19 epidemic, from 2.1 (95%CI: 1.6–2.7) to 1.4 (95%CI: 0.6–2.2) per 100,000 and from 9.0 (95%CI: 7.9–10.2) to 6.3 (95%CI: 2.9–9.7) per 100,000, respectively (Table 1). The RRR was 33.0% and 30.0% for salmonellaosis and campylobacteriosis, respectively, lower than the RRR observed for shigellosis.

**Figure 2:**
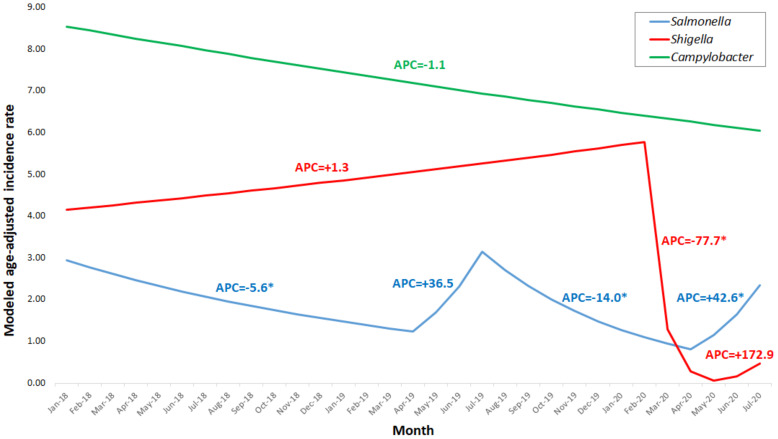
Modeled age-adjusted incidence rates of shigellosis, salmonellosis and campylobacteriosis per 100,000, January 2018–July 2020. * *p*-value < 0.05.

Figure 2 displays the modeled age-adjusted incidence rates of salmonellosis, shigellosis and campylobacteriosis per 100,000 between January 2018 and July 2020 calculated using Joinpoint software. 

Table 2 describes the annual percent change (APCs) observed for salmonellosis, shigellosis and campylobacteriosis between January 2018 and July 2020, also calculated by Joinpoint. For shigellosis, we have identified three trend periods in the modeled age-adjusted incidence rates: a non-significant increase between January 2018 and February 2020 (APC = 1.3), a significant, sharp decrease between February and May 2020 (APC = −77.7) and a non-significant increase between May and July 2020 (APC = 172.9) (Table 2). For salmonellosis, four trend periods were observed in the modeled age-adjusted incidence rates (Table 2): a significant decrease between January 2018 and April 2019 (APC = −5.6), a non-significant increase between April 2019 and July 2019 (APC = 36.5), a significant decrease between July 2019 and April 2020 (APC = −14.0) and a significant increase between April and July 2020 (APC = 42.6). For campylobacteriosis, a non-significant decrease was observed between January 2018 and July 2020 (APC = −1.1) (Table 2). 

## 4. Discussion

The purpose of the current study was to assess temporal trends of leading bacterial enteric diseases, salmonellosis, shigellosis and campylobacteriosis in Israel during the COVID-19 pandemic. We have shown that the relative risk reduction observed for shigellosis was higher (86.6%) than for salmonellosis (33.0%) and campylobacteriosis (30.0%). Using Joinpoint, we demonstrated a significant, sharp decrease in the incidence rate of shigellosis after COVID-19 onset. However, a significant decrease between July 2019 and April 2020 for salmonellosis was observed as well, but the change was not as sharp (APC = −14.0) as that observed for shigellosis (APC = −77.7) and can be explained by the seasonality of salmonellosis, as was also shown towards April 2019. 

Shigellosis is an enteric disease caused by bacteria of genus *Shigella* [4]. The symptoms presented in those infected are watery, bloody, or mucoid diarrhea; fever; stomach cramps and nausea [5], but asymptomatic infection may also occur [4]. The infectious dose is as low as 10 organisms [5], and transmission is possible via the fecal–oral route, including through direct person-to-person or sexual contact or indirectly through contaminated food, water or fomites [5]. We have previously shown that in Israel, the incidence of shigellosis is cyclic, characterized by incidence peaks occurring every 2–3 years [6].

There are several possible explanations for the significant, striking decrease in the incidence of shigellosis during the COVID-19 outbreak in Israel:

1. Social distancing is important in general in reducing the possibility of contact (shaking hands, touching fomites) which can enhance transmission of both respiratory and enteric pathogens such as SARS-CoV-2 and *Shigella*, respectively.
2. Hand hygiene, including washing hands with soap or using alcogel if washing is not possible, may prevent direct and indirect transmission of both SARS-CoV-2 and *Shigella* [7,8]. Khan et al. and Cohen et al. have shown that hand washing was inversely associated with the occurrence of *Shigella*, even in insanitary environments [7,8].
3. Environmental transmission of *Shigella* through fomites is highly applicable due to the low infectious dose required. Following the COVID-19 outbreak, the public awareness of fomite and surface sanitation increased dramatically, thus reducing the probability to be contaminated with *Shigella*.
4. Non-coronavirus patients avoided visiting their physician out of fear of contracting COVID-19, either from a healthcare worker or from a patient visiting the clinic. In Canada, a decrease observed in clinic visits due to code strokes was mainly associated with fear of being exposed to the SARS-CoV-2 but also with clinic referrals which were largely explained by hospital policies and the governmental lockdown [9]. The striking decrease observed in the incidence rate of shigellosis cannot be explained solely by the avoidance of visiting the clinic since we could expect a similar quantitative reduction in the incidence rate of both salmonellosis and campylobacteriosis, which was not observed. Thus, the reduction observed seems to reflect a truly lower rate of infection.
5. During the COVID-19 outbreak, doctor appointments were mostly performed by phone or video call. It has been shown that remote consultations effectively reduced the burden on hospitals, prevented overcrowding, reduced the risk of cross-infection, relieved patients’ anxiety during the COVID-19 outbreak and played an essential role in pandemic management [10]. However, physicians were less capable to clinically diagnose shigellosis and were less likely to send stool samples to the laboratory for confirmation. Since we count on data received from sentinel laboratories on isolates of *Shigella*, less available stool samples could have also contributed to the decrease in the incidence rate of culture-proven shigellosis, but this cannot be the sole explanation since a similarly enhanced decrease in culture-proven salmonellosis and campylobacteriosis could also have been expected.
6. The clinical laboratories equipment and personnel were all dedicated to testing for SARS-CoV2, and the option of neglecting other specimens testing cannot be ruled out. However, one could expect that the decrease observed in the identification of *Shigella* should have also been observed in the identification of *Salmonella* and *Campylobacter*, which was not the case.

Using our ongoing laboratory-based surveillance of bacterial enteric diseases, a similar effect of another respiratory pandemic on the drop in incidence of shigellosis was identified in April 2009 when the A(H1N1)pdm09 Influenza virus first appeared. The incidence of shigellosis remained low all long that year [6]. However, for salmonellosis and campylobacteriosis, no parallel decrease in the corresponding incidence rates in 2009 was observed [11,12].

A decrease in the occurrence of other infectious diseases transmitted from person to person during the COVID-19 pandemic, albeit respiratory diseases including seasonal respiratory virus infections was reported [13,14]. In China, the overall number of patients positive for influenza and other respiratory viruses during the COVID-19 period decreased significantly when compared with that in the same period of the last few two years [13]. Data extracted from the U.S. Centers for Disease Control and Prevention National Respiratory and Enteric Virus Surveillance System site revealed that influenza incidence was 8.7% in March 2020, compared with 25.0% in March 2019 [14]. It has been suggested that public health intervention, including social distancing, wearing masks and hand hygiene decreased significantly the incidence of respiratory infections [13,14]. 

To the best of our knowledge, our study is among the very few studies that examined changes in the incidence rates of culture-proven enteric diseases during the COVID-19 pandemic [15].

## 5. Conclusions

We have shown that during the COVID-19 epidemic, the incidence rate of shigellosis decreased significantly while a moderate decrease or no change in incidence were observed for other bacterial enteric diseases such as salmonellosis and campylobacteriosis, respectively. This decrease is most likely explained by the preventive measures applied to reduce the transmission of COVID-19, including social distancing and hand washing and was strongest for shigellosis, a disease that is mostly person-to-person transmitted, as compared to salmonellosis and campylobacteriosis which are mostly foodborne transmitted.

## Figures and Tables

**Table 1:** Mean age-adjusted incidence rates per 100,000 and risk reduction of shigellosis, salmonellosis and campylobacteriosis during March–July 2020 in comparison to March–July 2018–2019.

		Salmonellosis		Shigellosis		Campylobacteriosis	
			95%CI ^€^		95%CI ^€^		95%CI ^€^
March–July 2018–2019	aaIR ^£^	2.1	1.6–2.7	6.7	5.2–8.2	9.0	7.9–10.2
March–July 2020	aaIR ^£^	1.4	0.6–2.2	0.9	0.0–2.7	6.3	2.9–9.7
	RR	0.67	0.08–5.66	0.13	0.01–1.21	0.70	0.25–1.94
	RRR ^¥^ (%)	33.0		86.6		30.0	

^£^ aaIR—age-adjusted incidence rate. **^€^** CI—confidence interval. **^¥^** RRR—relative risk reduction.

**Table 2:** Trend periods for salmonellosis, shigellosis and campylobacteriosis between January 2018 and July 2020.

Period No.	Start Month	MAAIR ^¥^ per 100,000	End Month	MAAIR ^¥^ per 100,000	APC ^£^	*p*-Value
**Salmonellosis**						
1	January 2018	2.94	April 2019	1.24	−5.6	0.000252
2	April 2019	1.24	July 2019	3.15	36.5	0.321872
3	July 2019	3.15	April 2020	0.81	−14.0	0.000212
4	April 2020	0.81	July 2020	2.35	42.6	0.031211
**Shigellosis**						
1	January 2018	4.15	February 2020	5.77	1.3	0.318416
2	February 2020	5.77	May 2020	0.06	−77.7	0.032693
3	May 2020	0.06	July 2020	0.47	172.9	0.142301
**Campylobacteriosis**						
1	January 2018	8.54	July 2020	6.04	−1.1	0.086214

^¥^ MAAIR—modeled age-adjusted incidence rate per 100,000. ^£^ APC—annual percent change.

## Data Availability

All relevant data are within the manuscript.
